# Does providing atrial fibrillation patients, after pulmonary vein isolation, with a 1-lead ECG device relieve the emergency department?—A historically controlled prospective trial

**DOI:** 10.1371/journal.pdig.0000688

**Published:** 2024-12-20

**Authors:** Jasper L. Selder, Mark J. Mulder, Willem R. van de Vijver, Philip M. Croon, Leontine E. Wentrup, Stéphanie L. van der Pas, Jos W. R. Twisk, Igor I. Tulevski, Albert C. Van Rossum, Cornelis P. Allaart

**Affiliations:** 1 Amsterdam UMC, location Vrije Universiteit Amsterdam, department of Cardiology, Amsterdam Cardiovascular Sciences, Amsterdam, The Netherlands; 2 Amsterdam UMC, location University of Amsterdam, department of Cardiology, Amsterdam Cardiovascular Sciences, Amsterdam, The Netherlands; 3 Amsterdam UMC, location Vrije Universiteit Amsterdam, Epidemiology and Data Science, Amsterdam, The Netherlands; 4 Amsterdam Public Health, Methodology, Amsterdam, The Netherlands; 5 Cardiology Centers of the Netherlands, Amsterdam, The Netherlands; Iran University of Medical Sciences, ISLAMIC REPUBLIC OF IRAN

## Abstract

Atrial fibrillation (AF) is a prevalent and clinically significant cardiac arrhythmia, with a growing incidence. The primary objectives in AF management are symptom relief, stroke risk reduction, and prevention of tachycardia-induced cardiomyopathy. Two key strategies for rhythm control include antiarrhythmic drug therapy and pulmonary vein isolation (PVI), with PVI being recommended for selected patients. Even though PVI is effective, post procedural health care utilization is high, contributing to emergency department (ED) overcrowding, which is a global healthcare concern. The use of remote rhythm diagnostics, such as a 1-lead ECG device (KM), may mitigate this issue by reducing ED visits and facilitating more plannable AF care. *Objective*: This study aimed to assess whether providing AF patients with a 1-lead ECG device for 1 year after PVI would reduce ED utilization compared to standard care. Additionally, the study assessed whether this intervention would render AF care more plannable by reducing the incidence of unplanned cardioversions. *Methods*: A historically controlled, prospective clinical trial was conducted. The intervention group were patients undergoing PVI for AF between September 2018 and August 2020, all patients in this group received a 1-lead ECG device for 1 year for remote rhythm monitoring. The historical control group were patients undergoing PVI between January 2016 and December 2017; these patients did not receive a 1-lead ECG device. Data on ED visits, planned and unplanned cardioversions, and outpatient contacts in the year after the PVI were collected for both groups. *Results*: The study included 204 patients, 123 in the 1-lead ECG group and 81 in the standard care group. There was no statistically significant difference in the number of all-cause ED visits (63 vs 68 per 100 pts, respectively, p = 0.72), ED visits for possible rhythm disorders, or ED visits for definite rhythm disorders between the two groups. However, the 1-lead ECG group demonstrated a higher proportion of planned cardioversions compared to unplanned ones (odds ratio 4.9 [1.57–15.85], p = 0.007). *Conclusion*: Providing patients with AF following PVI with a 1-lead ECG device did not result in a statistically significant reduction in ED visits during the first year. However, it did improve the management of recurrent AF episodes by substituting unplanned cardioversions with scheduled ones.

Clinical Trials Registration Number NCT06283654.

## Author summary

In our study, we explored a new approach to manage atrial fibrillation, a disease that affects millions worldwide. Our goal was to see if using a simple handheld ECG device for monitoring heart rhythm could help patients avoid unnecessary visits to the emergency department (ED) after undergoing a common procedure known as pulmonary vein isolation (PVI). This procedure is often used to treat AF, but following it patients frequently visit the ED due to concerns about their heart rhythm, which can strain healthcare resources. We provided a group of patients with a 1-lead ECG device, which allows users to check their heart rhythm at any time. We compared the ED utilization over a year with that of patients who received standard care after PVI. Our hope was that by using the 1-lead ECG device, patients could better manage their condition from home and only seek medical help when truly necessary. Our findings showed that while the device did not statistically significantly reduce the number of ED visits overall, it did lead to a more planned approach to managing AF. Patients with the 1-lead ECG device had fewer unplanned treatments (cardioversions) for rhythm disorders, suggesting that the device helped to make more informed decisions about when hospital care was needed. Our research points to the potential benefits of remote monitoring technologies in managing AF and reducing the burden on emergency healthcare services.

## Introduction

### Atrial fibrillation and pulmonary vein isolation

Atrial fibrillation (AF) is the most prevalent sustained and clinically significant cardiac arrhythmia. Lifetime risk of developing AF is 22–24% at the age of 55 [1–2] and the prevalence of AF is estimated to double by 2060 [3–5]. Treatment of AF focuses on the relief of symptoms, reducing the risk of stroke and other arterial emboli and preventing cardiomyopathy caused by tachycardia. In order to relieve symptoms and prevent cardiomyopathy, a strategy of either rhythm or rate control can be chosen. Rate control consists of treatment with heart rate reducing drugs such as beta blockers. For rhythm control, pulmonary vein isolation (PVI) and antiarrhythmic drug therapy are the main strategies in order to prevent recurrence of AF. PVI is recommended for rhythm control where antiarrhythmic drugs fail and should be considered as first line therapy in selected patients [5]. In 2019, Andrade demonstrated, with an RCT comparing three commonly used AF ablation techniques, that 53.9% of AF patients were free from AF, atrial flutter and atrial tachycardia one year after ablation, with a burden reduction of over 98% [6].

### Emergency department crowding

Even though PVI is effective, post procedural health care utilization is high. Publications show that after PVI, 25–45% of the patients are seen on the emergency department (ED) at least once within 1 year, and 83% of these visits related to AF or atrial flutter [7,8]. Furthermore, when a cardioversion is needed, the ED length of stay can be up to 6 hours [9]. This contributes to crowding of the ED, resulting in ambulance diversion as well as complete ED admittance stops, which is considered a serious threat to the quality and availability of healthcare worldwide [10]. Using remote rhythm diagnostics for the post PVI patient could 1) prevent ED visits resulting in less ED crowding and 2) render AF care more plannable, especially in case of time-consuming cardioversions.

### AliveCor Kardia

The AliveCor Kardia Mobile (KM) is a device well suited for remote rhythm diagnostics, especially for AF. It comprises a small 1-lead ECG device connected to a smartphone with incorporated AF detection algorithm, connected to a cloud-based portal to store and access the ECGs. With the KM, patients can record their heart rhythm at any place, at any time, as many times as needed. Furthermore, the cardiologist can review the ECGs in the portal at any time [11].

### Study objective

The primary objective of this study was to assess whether handing out a 1-lead ECG device to AF patients for 1 year after PVI reduces ED utilization compared to standard care. The secondary objective was whether handing out a 1-lead ECG device to AF patients for 1 year after PVI would render AF care more plannable by reducing the incidence of unplanned cardioversions.

## Results

### Baseline characteristics

**Fig 1:**
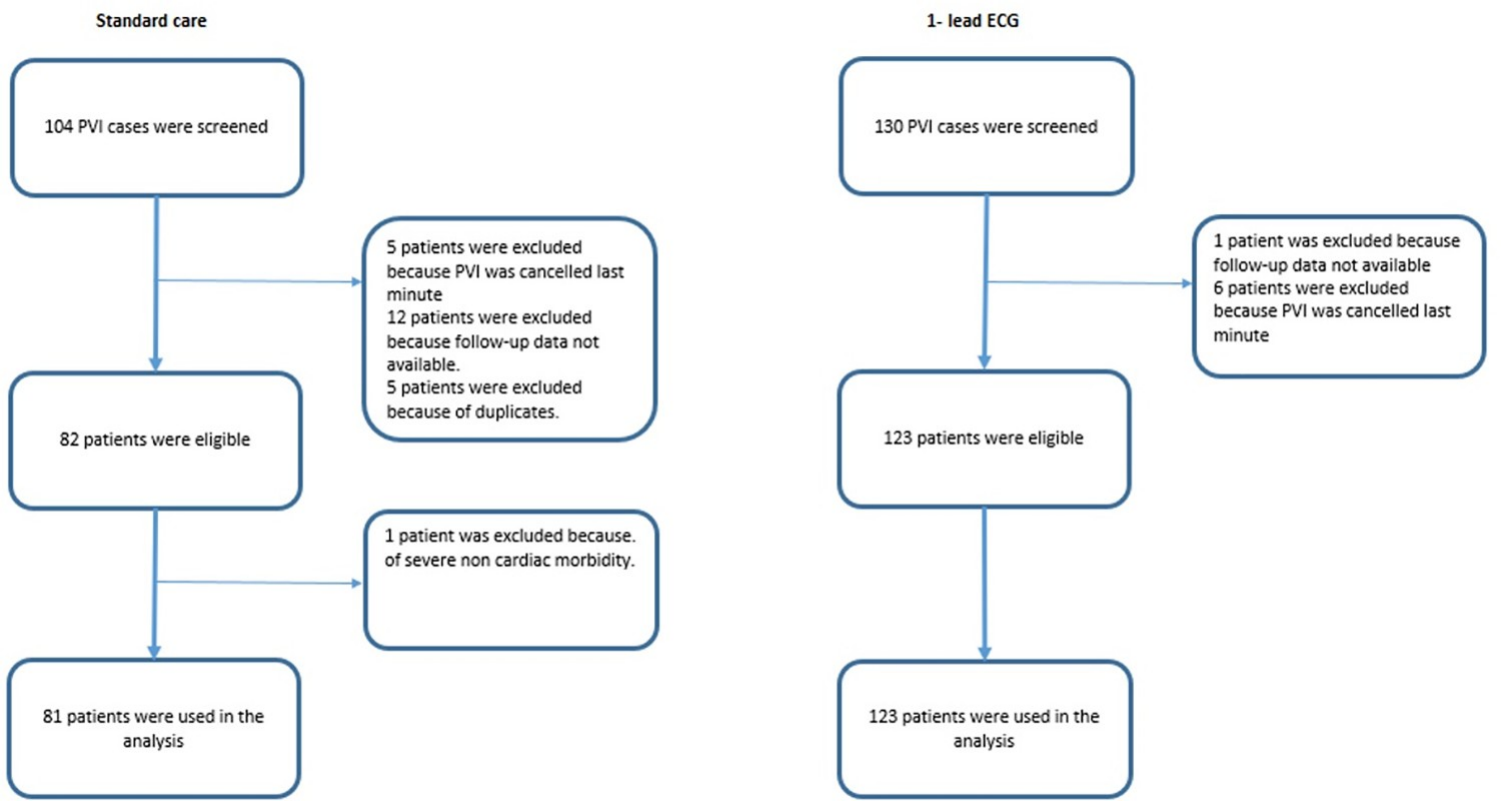
Flow diagram of the 1-lead ECG and the standard care cohort.

In total 204 patients were included, 123 patients in the 1-lead ECG group (prospective intervention cohort) and 81 patients in the standard care group (historical cohort), see the inclusion flow diagram, Fig 1. The average age was 63±10 years in both groups, with a BMI of 27±4 kg/m^2^ and a similar distribution of males (71% and 68%). There was no difference in the major comorbidities, except for previous stroke. Procedural complications occurred in 8% and 10%. The complete baseline characteristics of the two cohorts are shown in Table 1.

**Table 1:** Baseline patient and procedural characteristics of the 1-lead ECG and the standard care group (P value of the Fisher exact test or Mann Whitney U test).

	1-lead ECG group	Standard care group	P-value
Patient characteristics			
Mean age, y (SD)	63.4 (9.6)	63.0 (9.8)	0.722
Sex, No. Male	87 (70.7%)	55 (67.9%)	0.756
Hypertension	48 (39.0%)	26 (32.1%)	0.372
Diabetes mellitus	11 (8.9%)	5 (6.2%)	0.598
Heart failure	13 (10.6%)	7 (8.6%)	0.811
Previous stroke	6 (4.9%)	12 (14.8%)	0.021
Vascular disease	11 (8.9%)	10 (12.3%)	0.484
CHA2DS2-VASc, mean (SD)	1. 53 (1.3)	1.80 (1.5)	0.271
CHA2DS2-VASc 0	35 (28.5%)	22 (27.2%)	
CHA2DS2-VASc 1	31 (25.2%)	16 (19.8%)	
CHA2DS2-VASc 2	26 (21.1%)	15 (18.5%)	
CHA2DS2-VASc 3	21 (17.1%)	17 (21.0%)	
CHA2DS2-VASc > 3	10 (8.1%)	11 (13.6%)	
BMI, mean (SD)	26.61 (3.604)	27.16 (5.006)	0.403
Paroxysmal AF	78 (63.4%)	50 (61.7%)	1.000
Persistent AF	42 (34.1%)	29 (35.8%)	0.765
Long standing persisting AF	3 (2.4%)	1 (1.2%)	0.655
Procedural characteristics			
Additional ablation lines	24 (19.5%)	14 (17.3%)	0.716
Complications	10 (8.1%)	8 (9.9%)	0.802
Vasc access complications	5 (4.1%)	5 (6.2%)	
Hematoma	4 (3.3%)	5 (6.2%)	
Pseudo aneurysm	1 (0.8%)	0 (0.0%)	
Bradycardia	1 (0.8%)	2 (2.5%)	
Pleural effusion	1 (0.8%)	0 (0.0%)	
Pneumonia	0 (0.0%)	1 (1.2%)	
Pericardial effusion	3 (2.4%)	0 (0.0%)	

### Primary outcome: Emergency department visits

**Fig 2:**
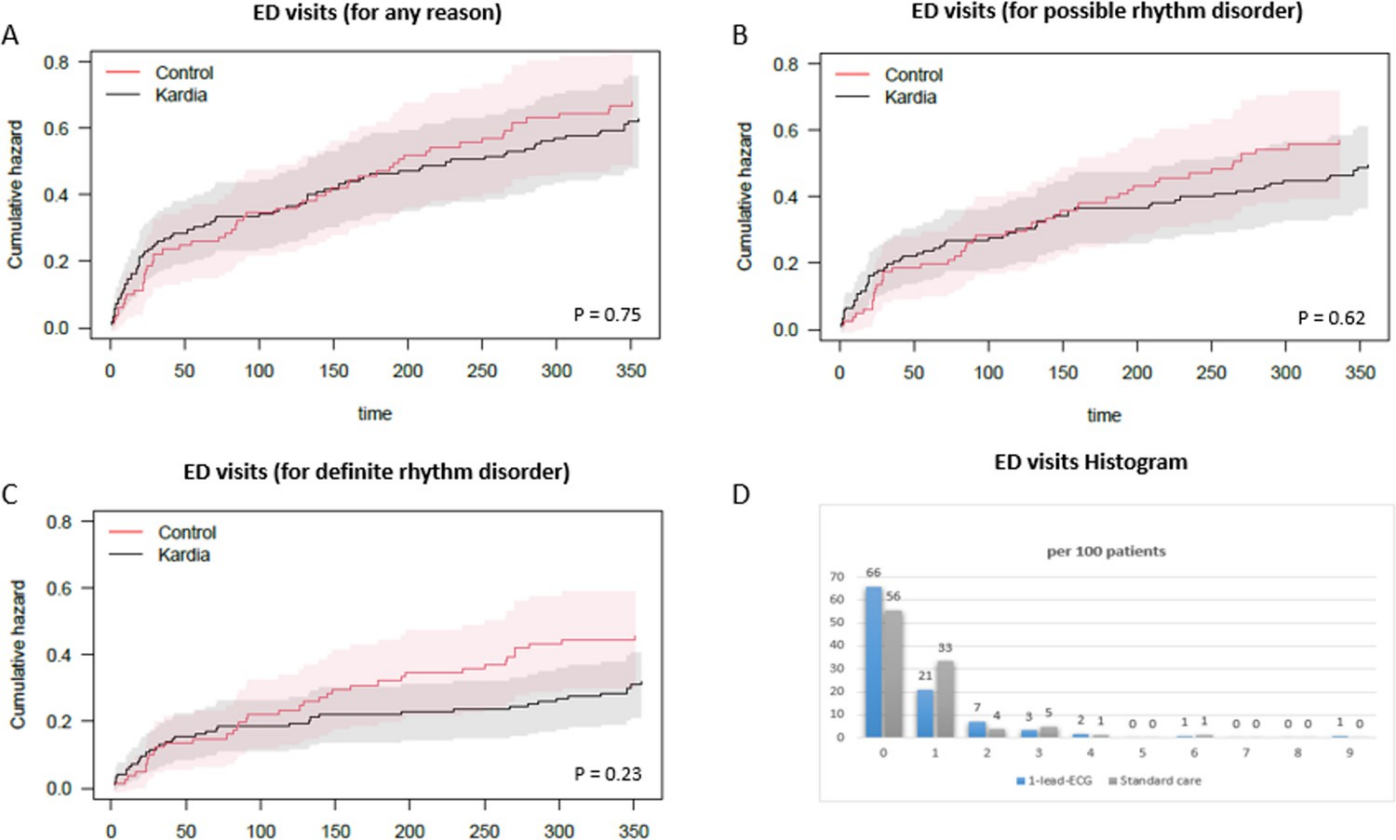
Cumulative Hazard and Histogram of Emergency Department (ED) Visits in the First Year Post-Ablation. This figure includes: (A) Cumulative hazard of all-cause ED visits, (B) Cumulative hazard of ED visits for possible rhythm disorders, (C) Cumulative hazard of ED visits for definite rhythm disorders, for both the 1-lead ECG (Kardia) group and the standard care (control) group, and (D) Histogram of all-cause ED visits. The red and grey areas depict the 95% confidence interval.

**Fig 3:**
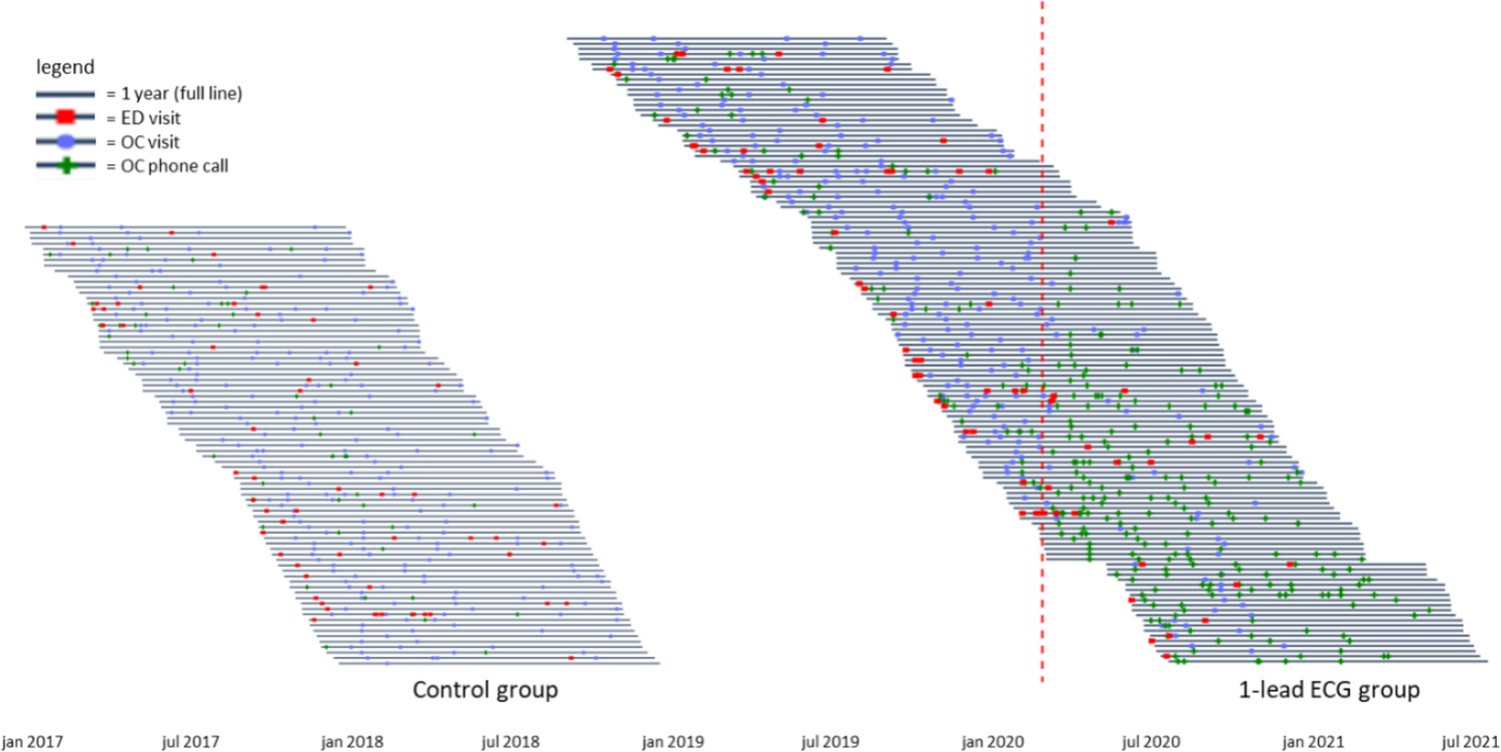
Timelines of the 1-year of follow up of all patients of both groups and their visits: ED Visits (red square), Outpatient Visits (blue dot), and Outpatient Phone Calls (green vertical line). In this figure, each line represents 1 year follow up of an individual patient. ED = Emergency Department, OC = Outpatient Clinic. The red dotted line indicates the onset of the COVID-19 Pandemic in The Netherlands.

There was no statistical difference in the primary outcome, the number of *all-cause* ED visits within 1 year after PVI between the two groups: 77 times in 123 patients (63 per 100 pts) in the 1-lead ECG group versus 55 times in 81 patients (68 per 100 pts) in the standard care group (p = 0.75). See Fig 2 for the cumulative hazard and the visit histogram. There was no statistical difference in the number of *possible rhythm disorder* ED visits (p = 0.62) and *definite rhythm disorder* ED visits (p = 0.23), (Table 2). The timelines of the ED visits and outpatient contacts, per group and per patient, are visualized in Fig 3. Sensitivity analyses (described in Methods) showed no relevant difference in outcome.

**Table 2:** Detailed ED visits characteristics for the 1-lead ECG group and the standard care group, per 100 patients. The first row is the primary outcome (total ED visits). First column is the absolute number of visits in the first year after PVI, and the second column is the number of visits per 100 patients. The first 3 rows include p-value for comparison (Marginal mean model—Wald test, see methods section).

	1-lead-ECG	N = 123	Standard care	N = 81	p-value
	*No*. *of visits*	*Per 100 patients*	*No*. *Of visits*	*Per 100 patients*	
Total ED visits	77	62.6	55	67.9	0.75
ED visits for possible rhythm disorder	60 (77.9%)	48.8	46 (83.6%)	56.8	0.62
ED visit for definite rhythm disorder	39 (50.6%)	31.7	37 (67.3%)	45.7	0.23
**Reason of emergency department visit**					
Palpitations	38 (49.4%)	30.9	35 (63.6%)	43.2	
Chest pain	12 (15.6%)	9.8	11 (20.0%)	13.6	
Dyspnoe	10 (13.0%)	8.1	0 (0.0%)	0.0	
Other	16 (20.8%)	13.0	9 (16.4%)	11.1	
Missing	1 (1.3%)	0.8	0 (0.0%)	0.0	
**Diagnosis of emergency department visit**					
Rhythm disorder	39 (50.6%)	31.7	37 (67.3%)	45.7	
Atrial fibrillation	19 (24.7%)	15.4	25 (31.6%)	30.9	
Atrial flutter	18 (23.4%)	14.6	12 (21.8%)	14.8	
Atrial tachycardia	2 (2.6%)	1.6	0 (0.0%)	0.0	
No rhythm disorder objectified (possible paroxysmal)	5 (6.5%)	4.1	3 (5.5%)	3.7	
Angina pectoris	3 (3.9%)	2.4	3 (5.5%)	3.7	
Myocardial infarction	1 (1.3%)	0.8	0 (0.0%)	0.0	
Decompensatio cordis	2 (2.6%)	1.6	0 (0.0%)	0.0	
Pericarditis	1 (1.3%)	0.8	0 (0.0%)	0.0	
Other	25 (32.5%)	20.3	12 (21.8%)	14.8	
Missing	1 (1.3%)	0.8	0 (0.0%)	0.0	
**Treatment of emergency department visit**					
Cardioversion	23 (29.9%)	18.7	27 (49.1%)	33.3	
Rate or rhythm medication	17 (22.1%)	13.8	6 (10.9%)	7.4	
Other	37 (48.1%)	30.1	22 (40.0%)	27.2	

### Secondary Outcome: Cardioversions

**Fig 4:**
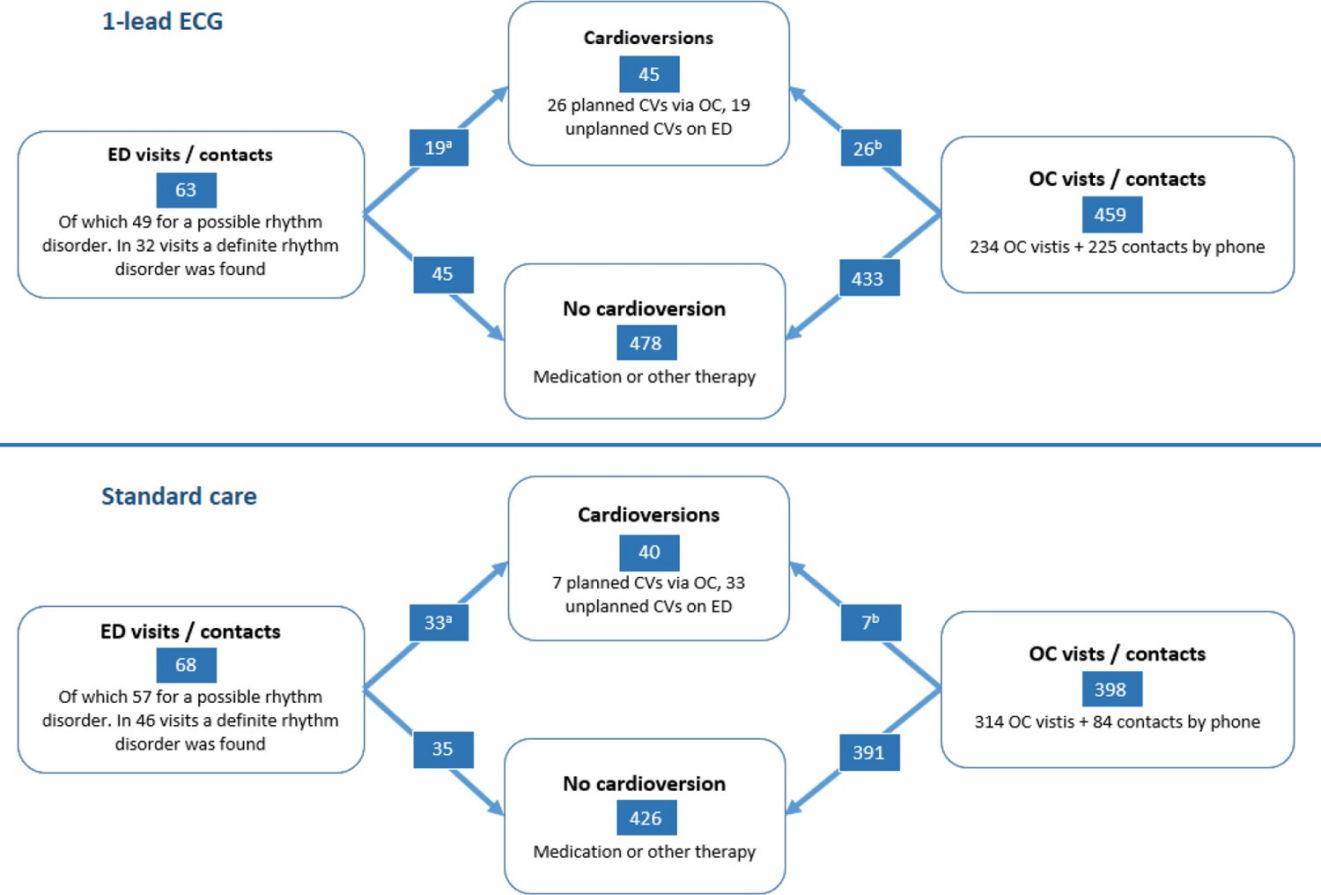
Secondary Outcome: The comparison of Cardioversions between the 1-lead ECG group and the standard care group. Left side: the unplanned pathways via the Emergency Department (ED). Right side: the planned pathway via the Outpatient Clinic (OC). All numbers are per 100 patients. a = number of unplanned cardioversions, b = number of planned cardioversions. The odds ratio of getting a *planned cardioversion* in the 1-lead ECG group versus the standard care group, was 4.99 [1.57–15.85] (p = 0.007).

The total number of cardioversions was similar in both groups, 45 versus 40 cardioversions per 100 patients. Taking into account the dependency of the observations within the patient, the odds ratio of getting a *planned cardioversion* versus an *unplanned cardioversion* in the 1-lead ECG group versus the standard care group, was 4.99 [1.57–15.85] (p = 0.007). Sensitivity analyses showed no relevant difference in outcome. For more details related to the cardioversions, see Fig 4.

### 1-lead ECG

During the 1 year follow up a total 10833 (8807 per 100 patients) 1-lead ECGs were recorded and send to our database. Average number of recordings per day was 30 (24 per 100 patients), with no significant difference between the weekdays. Peak recording time was between 8am - 11am. In total 2299 1-lead ECG recordings (21%) were classified as AF by the cardiologists, 8135 (75%) were normal sinus rhythm. For more detailed 1-lead ECG classifications and the agreement between the Kardia algorithm and the cardiologist see S1 Table.

## Discussion

### Main findings

In this study, providing patients with AF following PVI with a 1-lead ECG device did not result in a statistically significant reduction in ED visits during the first year. However, it did improve the management of recurrent AF episodes by substituting unplanned cardioversions with scheduled ones.

### ED visits

There was no significant difference in all-cause ED visits between the group using the 1-lead ECG device and the group receiving standard care. Several factors may account for this. Firstly, approximately one third of the ED visits were unrelated to rhythm disorders, rendering the 1-lead ECG device unhelpful in these cases. When considering only visits related to confirmed rhythm disorders, there appeared to be a slight reduction in the 1-lead ECG group (32/100 vs 46/100), though this difference did not reach statistical significance (p = 0.23). Secondly, we did not impose a strict protocol for the utilization of the 1-lead ECG device, and there was no dedicated 24/7 service center. This approach was taken assuming that such strict measures might be less likely to be adopted if the device had proven successful in reducing ED visits. A third factor to consider is the impact of the COVID-19 pandemic, which may have influenced ED visits. However, we conducted a sensitivity analysis that did not reveal any effect of COVID-19 on the ED visit data. Lastly, it is possible that the results from the 1-lead ECG device, rather than providing reassurance, may have prompted patients to visit the ED out of concern.

### Cardioversions

The lower frequency of emergency cardioversions observed in the 1-lead ECG group can potentially be elucidated through the following scenario. In the standard care group, patients likely reached out to the ED or outpatient clinic when experiencing palpitations. However, without a 1-lead ECG, it was challenging to assess the patients’ condition comprehensively, as both heart rhythm and frequency remained unknown. Consequently, many of these patients were advised to visit the ED for an ECG assessment. When AF/AFL was identified in the ED, emergency cardioversions were usually conducted directly on the ED. In contrast, within the 1-lead ECG group, heart rhythm and frequency were accessible during the initial phone contact. This probably led to situations where an immediate ED was not deemed necessary, such as when recurrent AF with an acceptable ventricular frequency was present. In these instances, an elective cardioversion could be scheduled, thereby averting the need for an emergency cardioversion. Therefore, we believe that the introduction of remote monitoring through a 1-lead ECG device has the potential to enhance patient management by allowing for timely and informed decision-making regarding AF episodes. Although the overall number of ED visits was not reduced, the shift from emergency to planned cardioversions could alleviate some strain on ED resources and improve patient outcomes by providing a more controlled treatment environment.

### Comparison with other studies

This study is the first to compare a cohort using a 1-lead ECG device with a cohort receiving standard care following PVI for AF. Our findings indicate high ED utilization during the first year post-PVI, with ED visit rates ranging from 63 to 68 per 100 patients. Specifically, there were 49 to 57 visits per 100 patients for suspected rhythm disorders and 32 to 46 visits per 100 patients for confirmed rhythm disorders. Several previous studies have examined ED utilization following PVI for AF, though none have specifically compared outcomes between patients using a 1-lead ECG device and those receiving standard care. In 2017, Biviano et al. reported 25 ED visits and 28 inpatient hospitalizations per 100 patients in the first year post-ablation [8]. This rate is lower than what we observed. Lopez et al. (2021) documented 51 ED visits per 100 patients within the initial 30 days post-PVI [12], suggesting a substantial early burden of ED utilization following the procedure. This finding aligns with our observation of high ED visit rates, particularly in the early post-PVI period. More recently, Friedman (2023) reported 16 ED visits per 100 patients within the initial 30 days post-PVI [13], which is lower than our rate. These variations in ED rates across studies may be attributed to differences in the study population, healthcare system, or post-ablation care protocols but emphasize the need for tailored approaches to monitoring and managing AF patients following PVI. The predominant reason for ED visits in these studies, as well as in our own, was palpitations, with the most common diagnosis of these ED visits being supraventricular tachycardia (SVT), including atrial fibrillation/flutter and other atrial tachycardias. These findings suggest that rhythm disturbances remain a significant concern for patients and healthcare providers alike in the post-PVI period. While direct comparisons across studies are challenging due to methodological differences, our findings contribute to the growing body of literature highlighting the substantial burden of ED visits following AF ablation procedures. Importantly, our study underscores the specific benefit of improving the scheduling of cardioversions through remote monitoring. This adds a nuanced understanding to the existing research, suggesting that while remote monitoring may not necessarily reduce the overall frequency of ED visits, it can streamline certain aspects of AF management. Furthermore, this study highlights a gap in the existing literature: the need for more comprehensive analyses of the impact of remote monitoring devices on long-term healthcare utilization and patient outcomes. Future studies should explore not only the frequency of ED visits but also the quality of care, patient satisfaction, and the overall cost-effectiveness of remote monitoring technologies in AF management.

### Limitations of this study

Despite our efforts to match baseline characteristics between the intervention and control groups, inherent biases may still exist due to the non-randomized study design. Additionally, relying on historical controls could introduce biases related to subtle changes in clinical practices or patient management over time. Furthermore, while we accounted for several key variables, there may be unmeasured confounders that could influence the outcomes. Factors such as variations in patient adherence to using the 1-lead ECG device, differences in healthcare access, and individual patient behaviors were not fully captured and could impact the results. Moreover, our findings may have limited generalizability beyond the specific population studied. The patients were recruited from multiple centers in the Amsterdam region (NL), and the results may not be applicable to other settings or populations with different characteristics.By addressing these limitations, we aim to provide a more comprehensive understanding of the study’s constraints and the necessary caution when interpreting the results.

### Conclusion and future perspective

Our study demonstrates that providing patients with atrial fibrillation (AF) after pulmonary vein isolation (PVI) with a 1-lead ECG device enhances the management of recurrent AF episodes, particularly by increasing the proportion of scheduled cardioversions over emergency ones. However, we did not observe a statistically significant reduction in emergency department (ED) visits during the first year post-PVI. These findings underscore the potential benefits of remote monitoring in certain aspects of AF management and lay the groundwork for further research to optimize its application in clinical practice.

## Material and Methods

### Study design and population

A historically controlled, prospective clinical trial was performed. The prospective intervention group consisted of consecutive consenting patients who underwent a first PVI for AF in the Amsterdam UMC–location VUmc, between September 2018 and August 2020. All patients from the intervention group received a KM during the follow-up period (for details see the following paragraph). The control group consisted of patients who underwent a first PVI in the Amsterdam UMC–location VUmc, between January 2016 and December 2017; they did not receive any form of remote rhythm monitoring. In both groups patients were referred for PVI from 4 hospitals in the region, the Amsterdam UMC–location VUmc, Amsterdam, Zaans Medical Centre, Zaandam, Noord West Clinics, Alkmaar and Cardiology Centres Netherlands (private clinic). In both groups, patients received comprehensive education from the AF physician assistant. This education covered the disease itself, self-management strategies, and how to respond to symptoms, particularly palpitations, which are the most common. Patients were guided on when it might be appropriate to watch and wait, and when to reach out to the outpatient clinic or the emergency department (ED). Furthermore, the education included cardiovascular risk management, emphasizing the importance of lifestyle adjustments to support cardiac health and manage AF effectively. The follow-up period was 1 year for both groups.

### Intervention group–Kardia mobile

Patients in the intervention group received a Kardia mobile (AliveCor, US) for remote rhythm monitoring. The KM device was connected to the Kardia smartphone application and the Kardia smartphone application was connected to a cloud based 1-lead ECG portal. When one finger of each hand was placed on the electrical pads of the KM, it started a 30-second recording, which was subsequently categorized by the algorithm of the Kardia app as either sinus rhythm, possible AF, unclassified or unreadable. The recordings with classifications were visible for the patient on the smartphone and uploaded automatically to a web portal, which was easily accessible for the treating physicians Participating patients were provided with the KM and encouraged to utilize it at their discretion. This meant they had the autonomy to use the device whenever they saw fit and as frequently as they considered necessary. They were informed that the KM’s recordings could serve as an additional resource for their treating physicians within the outpatient setting. During outpatient consultations, cardiologists had access to 1-lead ECG recordings through the web portal. All cardiologists in the 4 hospitals were made aware that the study’s protocol highlighted the importance of patient autonomy in self-monitoring while allowing cardiologists to exercise clinical discretion in their management approach. It was emphasized that the KM was designed as an ancillary tool to augment patient-centered care by facilitating both monitoring and informed decision-making in the management of cardiac arrhythmias. No extra nursing resources to monitor the 1-lead ECG data were allocated. Additionally, patients were reminded that using the device should not be considered a substitute for seeking immediate medical care in emergency situations. After the follow-up period, all recordings were analyzed by a team lead by two cardiologists, in case of disagreement a third cardiologist was decisive.

### Data collection and outcome measures

Baseline characteristics were extracted from the electronic medical record. Patient characteristics included age, gender, medical history, body mass index, and CHA2DS2-Vasc score. Procedural characteristics included the date of the procedure, initial ablation or repeat ablation, additional ablation lines, success of the ablation and acute complications. Data of all ED visits were collected including date, symptoms, diagnosis and treatment. A distinction was made between planned cardioversions and unplanned. A planned cardioversion was defined as a cardioversion that was scheduled through the outpatient clinic, either after a phone call or a physical consultation with the physician and was set for a moment in the future, as an elective procedure, at the first available cardioversion spot. An unplanned cardioversion meant that the patient arrived at the emergency department unplanned and underwent a cardioversion there directly. Furthermore, the number of OC visits and calls per patient were collected.

For the objectives the following outcome measures were used. For the primary objective: Number ED visits for any cause, number of ED visits for possible rhythm disorder (patients presenting with palpitations, dyspnea or chest pain, regardless of the definite diagnosis), number of ED visits for definite rhythm disorder (ED 12-lead ECG or 1-lead ECG with AF, atrial flutter or atrial tachycardia). For the secondary objective: Ratio between the number of planned cardioversions and the number of unplanned cardioversions.

### Statistical analysis

IBM SPSS version 26 (SPSS Inc., Chicago, IL, USA) and R version 4.1.0 (Project for Statistical Computing, GNU) were used to analyze the data. The power calculation was based on the assumption that ED visits followed a Poisson distribution, with a power of 80% and alpha of 5%, an estimated 10% unusable data registrations and an estimated 10% lost-to-follow-up. This resulted in a total of 130 patients that were necessary to detect a difference of 0.5 emergency department visits per patient per year. To account for the possibility of recurrent ED visits, marginal mean models were fitted, which do not require assumptions on time dependence between recurrent visits within an individual [14]. The models were fitted for the outcomes: time to visit for any reason, time to visit for possible rhythm disorder, time to visit for definite rhythm disorder. Significance of differences between the two patient groups was assessed with Wald-tests. Because the COVID-19 pandemic overlapped with the study period, sensitivity analyses were performed with only patients included up to 3, 6, or 9 months prior to 01-03-2020. To compare the 1-lead ECG group with the standard care group for getting a planned cardioversion versus an unplanned (emergency) cardioversion, logistic Generalized Estimating Equations (GEE-analysis) were used. GEE-analysis was used to take into account the dependency of the observations within the patient. For baseline characteristics and procedural characteristics, the Fisher exact test or the Mann-Whitney U test were used. For all statistical analyses p<0.05 was considered significant.

### Ethics Approval

Study Reference: VUmc 2018.539. Medical Ethics Review Committee of VU University Medical Center, which is registered with the US Office for Human Research Protections (OHRP) under IRB number IRB00002991. VU University Medical Center holds an FWA (Federal Wide Assurance) with the designation FWA00017598.

## Supporting information

S1 Checklist Strobe Condor Checklist. (DOCX)

S1 Protocol Clinical Trials Protocol. (PDF)

S1 Data Source data file (CSV). (CSV)

S1 Code R Code Statistics File. (RMD)

S1 Table Kardia Pro algorithm Evaluation. (DOCX)
