# Supplementary material for: Does providing atrial fibrillation patients, after pulmonary vein isolation, with a 1-lead ECG device relieve the emergency department?—A historically controlled prospective trial
Source: PLOS Digit Health. 2024 Dec 20;3(12):e0000688. doi: 10.1371/journal.pdig.0000688 (PMC11661629; doi:10.1371/journal.pdig.0000688)
Supplement: S1 Protocol — (PDF) [file pdig.0000688.s002.pdf]

**ClinicalTrials.gov PRS**
Last Update: 02/21/2024 06:28

**ClinicalTrials.gov ID: NCT06283654**

## Study Identification

Unique Protocol ID: VUmc 2018.539

Brief Title: Relieving the Emergency Department by Using a 1-lead ECG Device for Atrial Fibrillation Patients After Pulmonary Vein Isolation ( relievED )

Official Title: Relieving the Emergency Department by Using a 1-lead ECG Device for Atrial Fibrillation Patients After Pulmonary Vein Isolation: a Historically Controlled Prospective Trial

Secondary IDs:

## Study Status

Record Verification: February 2024

Overall Status: Completed

Study Start: September 30, 2018 [Actual]

Primary Completion: September 30, 2021 [Actual]

Study Completion: January 31, 2023 [Actual]

## Sponsor/Collaborators

Sponsor: Amsterdam UMC, location VUmc

Responsible Party: Principal Investigator
Investigator: Luuk Hopman [lhopman]
Official Title: prof dr
Affiliation: Amsterdam UMC, location VUmc

Collaborators:

## Oversight

U.S. FDA-regulated Drug: No

U.S. FDA-regulated Device: No

U.S. FDA IND/IDE: No

Human Subjects Review: Board Status: Not required

Data Monitoring: No

FDA Regulated Intervention: No

## Study Description

Brief Summary: In our study, we explored a new approach to manage atrial fibrillation, a disease that affects millions worldwide. Our goal was to see if using a simple handheld ECG device for monitoring heart rhythm could help patients avoid unnecessary visits to the emergency department (ED) after undergoing a common procedure known as pulmonary vein isolation (PVI). This procedure is often used to treat AF, but following it patients frequently visit the ED due to concerns about their heart rhythm, which can strain healthcare resources. We provided a group of patients with a 1-lead ECG device , which allows users to check their heart rhythm at any time. We compared the ED utilization over a year with that of patients who received standard care after PVI. Our hope was that by using the 1-lead ECG device, patients could better manage their condition from home and only seek medical help when truly necessary.

Detailed Description: Atrial fibrillation (AF) is a prevalent and clinically significant cardiac arrhythmia, with a growing incidence. The primary objectives in AF management are symptom relief, stroke risk reduction, and prevention of tachycardia-induced cardiomyopathy. Two key strategies for rhythm control include antiarrhythmic drug therapy and pulmonary vein isolation (PVI), with PVI being recommended for selected patients. Even though PVI is effective, post procedural health care utilization is high, contributing to emergency department (ED) overcrowding, which is a global healthcare concern. The use of remote rhythm diagnostics, such as a 1-lead ECG device, may mitigate this issue by reducing ED visits and facilitating more plannable AF care. Objective: This study aimed to assess whether providing AF patients with a 1-lead ECG device for 1 year after PVI would reduce ED utilization compared to standard care. Additionally, the study assessed whether this would enhance plannability of AF related healthcare utilization. Methods: A historically controlled, prospective clinical trial was conducted. The 'intervention' group are patients undergoing PVI for AF, all patients in this group receive a KM device for 1 year for remote rhythm monitoring. The historical control group were patients undergoing PVI between January 2016 and December 2017; these patients did not receive a KM. Data on ED visits, planned and unplanned cardioversions, and outpatient contacts in the year after the PVI were collected for both groups.

## Conditions

Conditions: Atrial Fibrillation

Keywords: 1-lead ECG
ED visits

## Study Design

Study Type: Observational

Observational Study Model: Other

Time Perspective: Prospective

Biospecimen Retention: None Retained

Biospecimen Description:

Enrollment: 204 [Actual]

Number of Groups/Cohorts: 2

## Groups and Interventions

| Groups/Cohorts | Interventions |
|---|---|
| 1-lead ECG group<br>Patients after PVI that received a 1-lead ECG | Device: 1-lead ECG<br>patient were handed out a 1 lead ECG device, they were free to use it anyway they wanted, even not at all |
| standard care group<br>standard care group | |

## Outcome Measures

Primary Outcome Measure:

1. ED visits
   [Time Frame: 1 year after PVI]

Secondary Outcome Measure:

2. ratio cardioversions emergency vs planned
   [Time Frame: 1 year after PVI]

## Eligibility

Study Population: Patients with all kinds of AF who underwent a PVI

Sampling Method: Probability Sample

Minimum Age: 18 Years

Maximum Age:

Sex: All

Gender Based: No

Accepts Healthy Volunteers:

Criteria: Inclusion Criteria:

- AF and PVI

Exclusion Criteria:

- <18y

## Contacts/Locations

Central Contact Person:

Central Contact Backup:

Study Officials:

Locations: **Netherlands**
Amsterdam UMC -vumc
Amsterdam, Netherlands
Contact: Jasper Selder

## IPDSharing

Plan to Share IPD:

## References

Citations:

Links:

Available IPD/Information:
