# Supplementary material for: Does providing atrial fibrillation patients, after pulmonary vein isolation, with a 1-lead ECG device relieve the emergency department?—A historically controlled prospective trial
Source: PLOS Digit Health. 2024 Dec 20;3(12):e0000688. doi: 10.1371/journal.pdig.0000688 (PMC11661629; doi:10.1371/journal.pdig.0000688)
Supplement: S1 Table — (DOCX) [file pdig.0000688.s005.docx]

| **Kardiapro algorithm** | **#** |  | **Cardiologist assessement** | **#** |
| --- | --- | --- | --- | --- |
| atrial fibrillation | 2561 |  | atrial fibrillation | 2039 |
|  |  |  | atrial flutter / SVT | 18 |
|  |  |  | sinus rhythm no extrasystoles | 83 |
|  |  |  | sinus rhythm with extrasystoles | 410 |
|  |  |  | unclassifiable | 10 |
| sinus rhythm | 6124 |  | atrial fibrillation | 4 |
|  |  |  | atrial flutter / SVT | 2 |
|  |  |  | sinus rhythm no extrasystoles | 4960 |
|  |  |  | sinus rhythm with extrasystoles | 1153 |
|  |  |  | unclassifiable | 6 |
| other | 605 |  | atrial fibrillation | 51 |
| (bradycardia or tachycardia, |  |  | atrial flutter / SVT | 140 |
| not otherwise defined) |  |  | sinus rhythm no extrasystoles | 331 |
|  |  |  | sinus rhythm with extrasystoles | 83 |
|  |  |  | unclassifiable | 0 |
| unclassifiable | 1543 |  | atrial fibrillation | 205 |
|  |  |  | atrial flutter / SVT | 145 |
|  |  |  | sinus rhythm no extrasystoles | 493 |
|  |  |  | sinus rhythm with extrasystoles | 622 |
|  |  |  | unclassifiable | 78 |
|  | 10833 |  |  | 10833 |

**S1 Table:** Classification of the 1-lead ECG recording by the Alivecor Kardia algorithm and subsequent classification by the two cardiologists.
